# Non-Union of Isolated Medial Condyle of Femur Hoffa Fracture: Case Report

**DOI:** 10.7759/cureus.34187

**Published:** 2023-01-25

**Authors:** Mukund Pai Manjeswar, Amit Kale, Harsh Raithatha, Shail Shah

**Affiliations:** 1 Orthopaedics, Dr. D. Y. Patil Medical College, Hospital and Research Centre, Pune, IND

**Keywords:** femur, fracture, hoffa, non-union, trauma

## Abstract

Isolated non-united Hoffa fracture of the femur is a rare finding. They are often missed due to the nature of the fracture and when not assessed appropriately. This is a case report of a 40-year-old male who encountered a high-velocity trauma; the fracture was probably missed on plain radiographs following the trauma. The patient presented to us eight months following the trauma with complaints of pain and decreased range of motion of his right knee (10 to 80 degrees of flexion) and the patient was unable to bear weight on the affected limb. On evaluation, the patient was found to have a non-united Hoffa fracture involving the medial condyle. The patient was treated with freshening of fracture followed by rigid fixation with cancellous screws and reconstruction plate. Postoperatively by week six, the patient achieved full range of motion and was able to walk without assistance with evidence of union on plain radiographs.

## Introduction

Fractures of the distal femur in the coronal plane involving the condyles are referred to as Hoffa fractures; they are often intra-articular and result from high-energy trauma [1,2]. Isolated Hoffa fractures account for less than 1% of fractures of the femur, with lateral condylar fractures accounting for over 75% of total cases [3]. Based on the system proposed by Letenneur, Hoffa fractures are classified into three types: I, II, and III, of which type I and III have intact soft tissue attachment while type II has compromised blood supply, thereby leading to increased risk of non union [4,5]. Non-union of Hoffa fracture is extremely rare. This is a case report of a non-union of a neglected Hoffa fracture involving the medial condyle, wherein bone grafting was done and partially threaded cancellous screws were used along with a reconstruction plate.

## Case presentation

**Figure 1:**
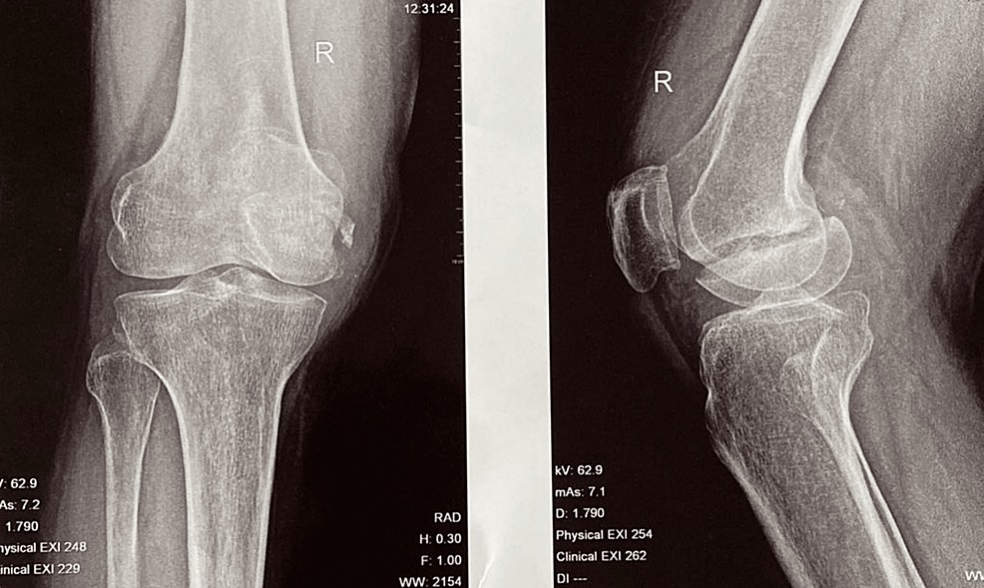
Plain radiograph of right knee in anteroposterior and lateral view showing Hoffa fracture of medial condyle

**Figure 2:**
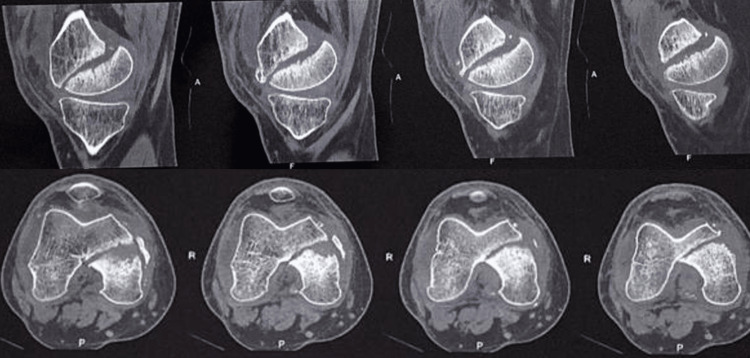
Computer tomography showing the medial condyle Hoffa fracture

**Figure 3:**
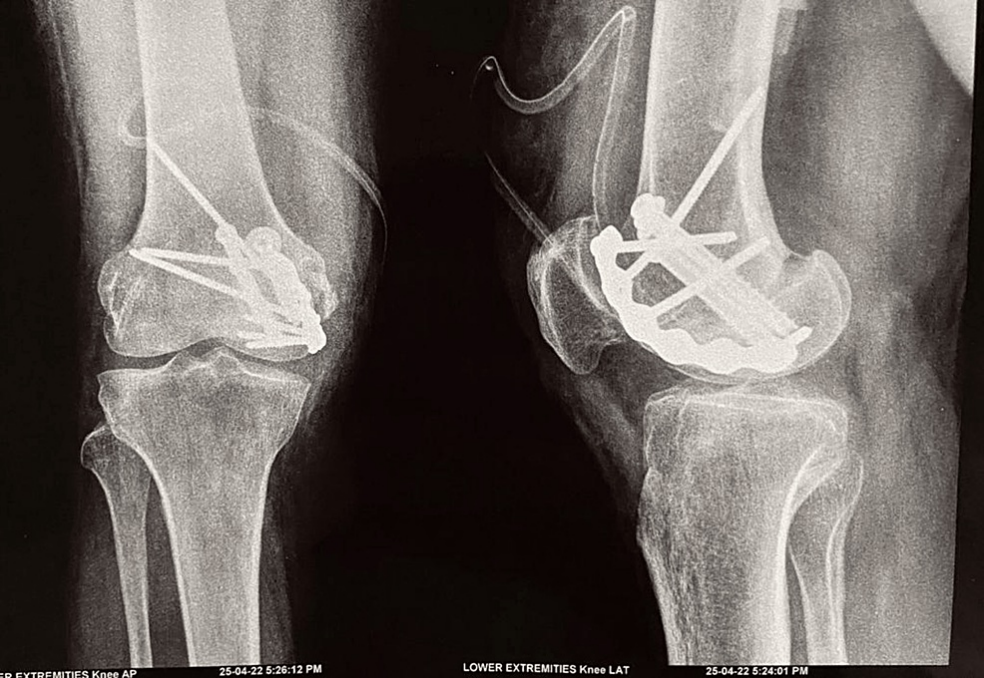
Post-operative plain radiograph of the right knee. Fracture fragment has been fixed using cancellous screws and reconstruction plate

A 40-year-old male presented with complaints of pain in his right knee with decreased range of motion. There was a history of fall from a motorbike (high-velocity trauma) eight months prior to his presentation, following which the patient went to a local hospital, where an undisplaced Hoffa fracture was probably missed on plain radiographs. His range of motion progressively decreased, affecting his daily routine activities, and he was unable to bear weight on the affected limb. On local examination of his right knee, there was no swelling. There was active range of motion of the knee from 10 to 80 degrees with a fixed flexion deformity of 10 degrees. Plain radiograph of the knee and CT confirmed the diagnosis of a non-united Hoffa fracture involving the medial condyle (Figures 1-2). The patient was taken up for fracture fixation after surgical clearance from the anaesthetist. The patient was positioned in supine position. An incision of 7cm was utilized over the antero-medial aspect of the distal femur, dissection was carried out until the non-united fragment was exposed. Fractured ends were freshened and curetted. Bone graft from iliac crest was used. Fracture fragment was fixed using thee 6.5mm partially threaded cannulated cancellous screws in the antero-superior to postero-inferior direction and a 3.5mm reconstruction plate. Post-operative radiograph was done of the right knee as seen in Figure 3.

**Figure 4:**
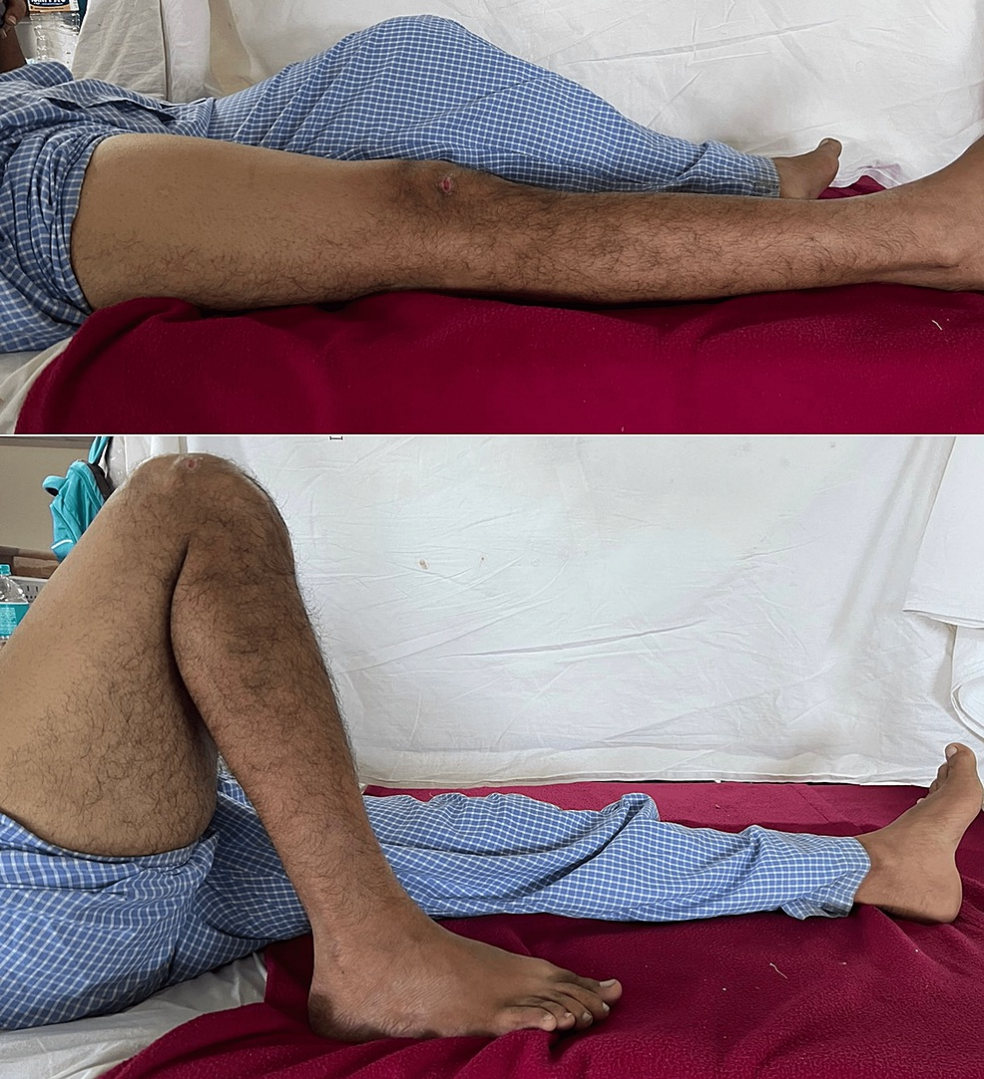
Range of motion of the right knee at post operative week six with full extension and flexion up to 130 degrees

The patient was immobilised in an above-knee slab for two weeks with elevation. Cefuroxime injection was given as antibiotic cover along with tramadol injection for pain control. Sutures were removed by the 15th post-operative day. Static quadriceps and hamstring exercises were started after suture removal along with range of motion of the knee up to 60 degrees. After the 3rd post-operative week, knee flexion was increased up to 100 degrees with walker-assisted partial weight-bearing walking, 130 degrees by the end of the 4th post operative week along with full-weight bearing-walking. The patient was able to walk without the help of a walker by the 6th week and achieved full range of motion of the knee (Figure 4). 

## Discussion

Non-union of Hoffa fracture is rare and requires adequate fixation. Undisplaced Hoffa fractures are often missed and when neglected, can lead to non-union. An antero-medial incision is preferred in order to adequately expose the fracture fragment [6,7]. A non-operative intervention leads to the risk of avascular necrosis, arthritis with articular incongruity, and non-union [5,8]. In our study, the reason for non-union can be contributed due to soft tissue interposition along with the synovial fluid antagonistic effect on bone formation. Patel et al. suggested an anterior approach for the fixation of the Hoffa fragment [3]. Various studies have suggested the need for freshening of fracture edges and rigid internal fixation with interfragmentary compression [4,7-10]. When it comes to the method of fixation, it has been found that the use of cancellous 6.5mm screws is superior when compared to 4mm screws, and that placement of the screws in antero-posterior direction prevents the breakage of the head of the screw as it does not come into the articular surface [9,11,12]. In our case, utilising a reconstruction plate in addition to 6.5mm cancellous screws provided a better stable fixation. We were able to achieve good post-operative results which were reflected by the patient's full range of motion of the operated knee by the 6th week.

## Conclusions

Hoffa fracture of the medial condyle with non-union is a rare finding. It requires adequate reduction and fixation in order to restore the function of the knee. Using a reconstruction plate with cancellous cannulated screws has proved to be efficient in this case as it helped restore the range of motion of the knee and the weight-bearing function of the knee.
